# Variant histology in urothelial carcinoma: associated factors and survival impact after radical cystectomy

**DOI:** 10.1186/s12894-026-02232-z

**Published:** 2026-06-23

**Authors:** Emre Uzun, Serkan Ozen, Samet Senel, Hasan Batuhan Arabaci, Antonios Koudonas, Huseyin Gultekin, Yalcin Kizilkan, Cavit Ceylan, Cuneyt Ozden

**Affiliations:** 1 Department of Urology, Ankara City Hospital, Üniversiteler Mahallesi 1604. Cadde No: 9, Çankaya, Ankara, Turkey; 2 First Department of Urology, School of Medicine, Aristotle University of Thessaloniki, Thessaloniki, Greece

**Keywords:** Bladder cancer, Radical cystectomy, Urothelial carcinoma, Variant histology

## Abstract

**Purpose:**

To investigate the factors associated with variant histology (VH) and prognostic significance of VH in urothelial carcinoma (UC) among patients undergoing radical cystectomy (RC) with ileal conduit urinary diversion.

**Methods:**

A retrospective analysis was conducted on 317 patients who underwent RC with ileal conduit diversion between 2019 and 2025. Patients were categorized based on the presence of VH in the final pathology. Demographic, clinical, perioperative, and pathological data were compared. Logistic regression was performed to identify factors associated with VH, while Kaplan–Meier and uni-multivariable Cox regression analyses evaluated overall survival (OS) and cancer-specific survival (CSS).

**Results:**

VH was detected in 139 patients (43.8%), with squamous differentiation being the most prevalent variant, observed in 80 patients of the 317 patients who underwent RC with ileal conduit urinary diversion. Presence of hydronephrosis, clinical lymph node positivity and WBC > 7695 g/dl were defined as factors associated with VH in multivariate logistic regression analysis. Kaplan–Meier survival analysis illustrated a statistically significant reduction in OS and CSS among patients harboring VH compared to those with pure UC following RC and ileal conduit urinary diversion (53.8 vs. 36.8 months for OS and 68 vs. 53 months for CSS, log-rank test, *p* < 0.001). Multivariable Cox regression analysis revealed that presence of variant histology was associated with reduced OS (HR = 1.624; 95% CI = 1.094–2.411; *p* = 0.016) and CSS (HR = 1.945; 95% CI = 1.043–3.626; *p* = 0.036).

**Conclusion:**

VH in UC is associated with advanced disease and poorer survival outcomes following RC. Certain preoperative factors—hydronephrosis, clinical lymphadenopathy, and elevated WBC—may be associated with the presence of VH. Early recognition of these features could guide individualized treatment strategies and improve prognostic assessment. However, the retrospective and single-center nature of this study may introduce selection and information bias, and the findings should be interpreted with caution until validated by prospective multicenter trials.

## Introduction

Bladder cancer (BCa) represents the seventh most frequent cancer globally, and its development has been etiologically associated with several factors, such as smoking, occupational exposure to chemicals, and radiation. Both incidence, prevalence and BCa-specific death are up to three times higher in the male patients, while the statistical projections for the next 20 years foresee a gradual reduction of the above gap among men- women, since the number of deaths among men is expected to increase up to 54% in contrast to an increase of 200–300% for women [1].

Since the management of BCa is mostly based on the tumor pathology in various clinical scenarios, the optimization of pathological reports is of utmost importance to achieve a precise and standardized depiction of tumor biology and its inherent risk for the patient. This optimization is translated into continuous updates of pathological reporting, and recently into classification by molecular markers, or artificial intelligence (AI) methods [2]. One of the topics that attracts the attention of the specialized urological pathologists is the finding of secondary histological subtypes in the specimens of urothelial BCa.

In general, urothelial carcinoma (UC) is recognized for including a wide spectrum of microscopic features and respective biological behaviours, which are matched to different histological entities. More precisely, two-thirds of urothelial BCa are characterized as pure UC, or conventional carcinoma, while one-third of BCa specimens demonstrate the presence of variant histology (VH). The latest includes several subtypes, such as nested, micropapillary, plasmacytoid, clear cell, and other variants [3]. Since the prognostic implications of the above variants are not certain, the recognition and reporting of their presence in the histological report is necessary to unveil their effect on patient survival [3]. Evidence derived from T1 urothelial BCa extracted by transurethral resection of bladder tumor (TURBT), there are indications that urothelial variants do not demonstrate a homogenous biological behaviour, and there are VH that are associated with high-risk (micropapillary, nested, glandular), or low-risk (squamous, microcystic, inverted growth) disease [4].

Regarding the effect of VH on the prognosis of patients treated with radical cystectomy (RC), the existing evidence is inconclusive, while the emergence of new pharmaceutical treatments in the neoadjuvant or adjuvant setting renders the recognition of VH at the earliest time point necessary [5]. In 2022, Ogbue et al. reported that neoadjuvant chemotherapy (NAC) is meaningful for patients with VH, yet the effect of NAC on the different subtypes is unclear [6]. Another study by Speir et al. evaluated the effect of NAC on urothelial BCa with squamous variant and reported that NAC was associated with higher rates of downstaging and organ-confined disease at cystectomy, especially for the tumors with < 50% involvement by the squamous variant [7]. Similarly, the significance of VH in urothelial BCa was underlined in another study by Lobo et al., who reported that chemotherapy is particularly effective in small cell carcinoma and lymphoepithelioma-like carcinoma. Nevertheless, the authors of the above study stated that the diagnosis of VH may be problematic due to sampling limitations and interobserver variability [8]. In 2021, Lonati et al. compared the accuracy of TURBT in detecting variant urothelial tumors, and in their conclusions, they stated that TURBT seems to have a poor to moderate diagnostic ability, and additional methods are needed to reliably identify the presence of VH before cystectomy [9].

From the above evidence, it can be extrapolated that the pre-cystectomy prediction of VH may be clinically relevant for the patient, and the histological examination of the TURBT specimen is not adequately precise for the timely recognition of urothelial variants. Primary objective of this study was to unveil the factors that are significantly associated with the presence of VH. Secondary objective was reveal the effect of VH on the cancer-specific survival (CSS) and overall survival (OS) of the patients.

## Materials and methods

### Study design and patient selection

A retrospective evaluation was conducted on the medical records of all consecutive 386 patients who underwent RC with ileal conduit urinary diversion for UC between February 2019 and May 2025. All surgical interventions were performed by surgeons with extensive experience in the field. Patients with incomplete clinical data (*n* = 69) and those diagnosed with non-urothelial bladder malignancies were excluded from the analysis. The 69 excluded cases primarily lacked postoperative pathological or follow-up data. After these exclusions, 317 patients with complete datasets were included in the final analysis. This study received ethical approval from the Institutional Review Board of Ankara City Hospital (approval number: TABED 1-25-1512).

The demographic (age, gender and body mass index [BMI], preoperative (previous surgery history, intravesical treatment history, NAC history, concomitant malignancy, presence of ascites, presence of hydronephrosis, smoking status, American Society of Anesthesiologists classification [ASA] score, functional capacity, clinical T stage, clinical grade, clinical lymph node positivity, presence of distant metastasis, hemoglobin, white blood cell [WBC], serum creatine, blood nitrogen urea, serum albumin, sodium and potassium levels, comorbidities), intraoperative (operation duration, incision length, urinary diversion technique, length of bowel segment used in urinary diversion, amount of bleeding, amount of blood transfusion, duration of total parenteral nutrition administration) and postoperative (hospitalization, time to oral nutrition, ureteral catheterization duration, complications, pathological T stage, pathological N stage, presence of VH, number of excised lymph nodes, number of metastatic lymph nodes, presence of carcinoma in situ (CIS), presence of prostate invasion, and concomitant primary prostate cancer, follow-up period, OS, CSS) characteristics of patients were collected from hospital database. The patients were classified into two main groups according to the presence of VH in the final pathology as those with one or more histological variants (VH group) and those without any variant components (pure UC group). In cases where more than one variant subtype was identified within the same specimen, the patient was categorized in VH and included as a single VH case. No subgroup analysis was performed according to the specific type or number of variant components. A comparison was made between the two groups.

Postoperative complications were evaluated according to the Clavien-Dindo classification system [10]. The VH of UC was determined according to the World Health Organization (WHO) classification [11]. The tumor stage was determined according to the current TNM classification of BCa [12]. OS was calculated from the date of RC and ileal conduit urinary diversion operation until death from any cause or the end of follow-up period. CSS was calculated from RC and ileal conduit urinary diversion operation to death due to UC or the end of follow-up period.

### Statistical analysis

Data coding and statistical analyzes were performed on the computer using the SPSS 22 software package program (IBM SPSS Statistics, IBM Corporation, Chicago, IL). The normality of the variables was assessed using the Shapiro-Wilk test. Normally distributed variables were expressed as mean ± standard deviation, while non-normally distributed variables were expressed as median (interquartile range [IQR]). For non-categorical variables, the independent samples T test or Mann Whitney U test was used. For categorical variables, Chi-square or Fisher’s exact tests were used. In comparative analysis, multivariate logistic regression analyses with the Backward LR method were used to evaluate the effectiveness of preoperative characteristics associated with the presence of VH, where a significant difference was found between the pure UC and VH groups. The cut-off values for parameters associated with VH were analyzed with the receiver operating characteristic (ROC) curve at 95% confidence interval (CI). Kalpan–Meier survival curves were constructed for the patients with pure UC and VH. We analyzed the difference in OS and CSS between these two groups of patients using the log rank test. To identify factors including presence of VH associated with OS and CSS, Uni-multivariable Cox regression analyses were performed. In the univariable analysis, each covariate was first tested. Variables with a p value less than 0.10 in the univariable analysis, were subsequently included in the multivariable model individually with Bacward LR method to adjust for confounders. Variables representing overlapping or collinear clinical features that could influence each other’s significance were not included together in the multivariable analysis. In accordance with the rule of one variable per ten observed events, the final multivariable model was constructed to avoid model overfitting. Cases with a p-value below 0.05 were considered statistically significant.

## Results

The mean age of the 317 patients who underwent RC with ileal conduit urinay diversion was 65.8 ± 7.8 years. The majority of the cohort were male (*n* = 279, 88%). Clinical staging revealed that 222 patients (70%) were classified as T2, while 74 patients (23.3%) exhibited clinical lymph node positivity. The median operative time was 360 min (IQR = 300–420). Postoperative complications occurred in 141 patients (44.5%). The median number of lymph nodes excised was 16 (IQR = 12–23). Prostate invasion was identified in 17 patients (5.4%) (Table 1). VH was detected in 139 patients (43.8%), with squamous differentiation being the most prevalent variant, observed in 80 patients (Table 2).

**Table 1:** Comparative analysis of demographic, preoperative, intraoperative, and postoperative parameters of patients undergoing radical cystectomy with ileal conduit diversion for urothelial carcinoma

	Total (*n* = 317)	Pure Urothelial Carcinoma (*n* = 178, 56.2%)	Variant Histology (*n* = 139, 43.8%)	*p*
Demographic characteristics				
Age (year) (Mean ± SD)	65.8 ± 7.8	66.3 ± 7.3	65.1 ± 8.5	0.267^m^
Male gender, n (%)	279 (88)	156 (87.6)	123 (88.5)	0.817^c^
BMI (kg/m^2^) (Ortalama ± SD)	26.2 ± 3.8	26.3 ± 4	26 ± 3.5	0.228^t^
Preoperative characteristics				
Previous surgery history, n (%)	54 (17)	33 (18.5)	21 (15.1)	0.42^c^
Intravesical treatment history, n (%)	34 (10.7)	25 (14)	9 (6.5)	**0.031** ^**c**^
Neoadjuvant chemotherapy history, n (%)	60 (18.9)	37 (20.8)	23 (16.5)	0.339^c^
Concomitant malignancy, n (%)	13 (4.1)	8 (4.5)	5 (3.6)	0.689^c^
Presence of ascites, n (%)	1 (0.3)	0 (0)	1 (0.7)	
Presence of hydronephrosis, n (%)	138 (43.5)	66 (37.1)	72 (51.8)	**0.009** ^**c**^
Smoking status, n (%)	285 (89.9)	160 (89.9)	125 (89.9)	0.991^c^
ASA score				
1, n (%)	6 (1.9)	4 (2.2)	2 (1.4)	0.664^f^
2, n (%)	162 (51.1)	94 (52.8)	68 (48.9)	
3, n (%)	142 (44.8)	75 (42.1)	67 (48.3)	
4, n (%)	7 (2.2)	5 (2.9)	2 (1.4)	
Functional capacity				
Independent, n(%)	310 (97.8)	174 (97.8)	136 (97.8)	0.634^f^
Partially-dependent, n (%)	7 (2.2)	4 (2.2)	3 (2.2)	
Dependent, n (%)	0 (0)	0 (0)	0 (0)	
Clinical T stage				
Ta, n (%)	16 (5)	11 (6.2)	5 (3.6)	0.33^c^
T1, n (%)	74 (23.4)	45 (25.3)	29 (20.9)	
T2, n (%)	227 (71.6)	122 (68.5)	105 (75.5)	
Primary MIBC	44 (19.4)	21 (17.2)	23 (21.9)	0.373^c^
NMIBC-to-MIBC	183 (80.6)	101 (82.8)	82 (78.1)	
Clinical grade				
Grade 1, n (%)	12 (3.8)	7 (3.9)	5 (3.6)	0.877^c^
Grade 2, n (%)	0 (0)	0 (0)	0 (0)	
Grade 3, n (%)	305 (96.2)	171 (96.1)	134 (96.4)	
Clinical lymph node positivity, n (%)	74 (23.3)	29 (16.3)	45 (32.4)	**0.001** ^**c**^
Presence of distant metastasis, n (%)	21 (6.6)	10 (5.6)	11 (7.9)	0.415^c^
Hemoglobin (g/dl) (Mean ± SD)	12.5 ± 1.9	12.6 ± 1.9	12.3 ± 2	0.368^t^
WBC (g/dl) (Mean ± SD)	8608.6 ± 3368.8	7878 ± 2487.8	9544.3 ± 4059.3	**< 0.001** ^**m**^
Serum creatine (mg(dl) (Mean ± SD)	1.1 ± 0.4	1.1 ± 0.4	1.1 ± 0.4	0.066^m^
Blood nitrogen urea (mg(dl) (Mean ± SD)	19.6 ± 7	18.9 ± 6.4	20.5 ± 7.6	0.095^m^
Serum albumin (gr/l) ((Mean ± SD)	41.5 ± 4.8	41.7 ± 4.5	41.2 ± 5.2	0.738^m^
Sodium (mEq/l) (Mean ± SD)	139.6 ± 2.7	139.8 ± 2.7	139.3 ± 2.7	0.125^m^
Potassium (mEq/l) (Mean ± SD)	4.4 ± 0.4	4.4 ± 0.5	4.4 ± 0.4	0.727^m^
Comorbidities				
Coronary artery disease, n (%)	47 (14.8)	28 (15.7)	19 (13.7)	0.608^c^
Heart failure, n (%)	15 (4.7)	7 (3.9)	8 (5.8)	0.448^c^
HT, n (%)	137 (43.2)	74 (41.6)	63 (45.3)	0.504^c^
DM, n (%)	75 (23.7)	37 (20.8)	38 (27.3)	0.173^c^
Thyroid disease, n (%)	13 (4.1)	8 (4.5)	5 (3.6)	0.682^c^
CKD, n(%)	138 (43.5)	70 (39.3)	68 (48.9)	0.087^c^
CVD, n(%)	15 (4.7)	5 (2.8)	10 (7.2)	0.068^c^
COPD, n(%)	37 (11.7)	22 (12.4)	15 (10.8)	0.666^c^
Intraoperative characteristics				
Operation duration (minutes) (Mean ± SD)	366.3 ± 85.8	366.6 ± 88.9	366 ± 81.9	0.99^m^
Incision length				
Infraumbilical midline, n (%)	142 (44.8)	71 (39.9)	71 (51.1)	**0.047** ^**c**^
Supra and infraumbilical midline, n (%)	175 (55.2)	107 (60.1)	68 (48.9)	
Urinary diversion technique				
Bricker, n (%)	188 (59.3)	100 (56.2)	88 (63.3)	0.2^c^
Wallace, n (%)	129 (40.7)	78 (43.8)	51 (36.7)	
Length of bowel segment used in urinary diversion (cm) (Mean ± SD)	19.9 ± 4.7	20 ± 5.3	19.7 ± 3.7	0.806^m^
Amount of bleeding (mL) (Median) (IQR)	800 (500–1275)	800 (500–1250)	800 (500–1300)	0.853^m^
Amount of blood transfusion (Unit) (Median) (IQR)	0 (0–1)	0 (0–1)	0 (0–1)	0.159^m^
Duration of TPN administration (days) (Median) (IQR)	0 (0–4)	0 (0–4)	0 (0–5)	0.647^m^
Postoperative characteristics				
Hospitalization (days) (Median) (IQR)	15 (12-19.5)	15 (12–20)	15 (12–19)	0.668^m^
Time to oral nutrition (days) (Median) (IQR)	3 (2–4)	3 (2–4)	3 (2–4)	0.591^m^
Ureteral catheterization duration (days) (Median) (IQR)	19 (10.5–21)	20 (10–21)	15 (11–21)	0.983^m^
Complications (Clavien-Dindo classification system), n (%)	141 (44.5)	73 (41)	68 (48.9)	0.16^c^
Grade 1, n (%)	64 (20.2)	34 (19.1)	30 (21.6)	0.55^f^
Grade 2, n (%)	3 (0.9)	1 (0.6)	2 (1.4)	
Grade 3a, n (%)	43 (13.6)	22 (12.3)	21 (15.1)	
Grade 3b, n (%)	21 (6.6)	11 (6.2)	10 (7.2)	
Grade 4a, n (%)	2 (0.6)	2 (1.1)	0 (0)	
Grade 4b, n (%)	2 (0.6)	0 (0)	2 (1.4)	
Grade 5, n (%)	6 (1.9)	3 (1.7)	3 (2.2)	
Pathological T stage				
Tis, n (%)	9 (2.8)	8 (4.5)	1 (0.7)	0.632^f^
T0, n (%)	33 (10.4)	33 (18.5)	0 (0)	
Ta, n (%)	8 (2.5)	8 (4.5)	0 (0)	
T1, n (%)	21 (6.6)	18 (10.1)	3 (2.2)	
T2a, n (%)	20 (6.3)	12 (6.7)	8 (5.8)	
T2b, n (%)	50 (15.9)	32 (18)	18 (12.9)	
T3a, n (%)	58 (18.3)	24 (13.5)	34 (24.5)	
T3b, n (%)	60 (18.9)	22 (12.4)	38 (27.3)	
T4a, n (%)	55 (17.4)	21 (11.8)	34 (24.5)	
T4b, n (%)	3 (0.9)	0 (0)	3 (2.2)	
Pathological N stage				
N0, n (%)	226 (71.3)	142 (79.8)	84 (60.4)	**0.001** ^**c**^
N1, n (%)	36 (11.3)	16 (9)	20 (14.4)	
N2, n (%)	45 (14.2)	18 (10.1)	27 (19.4)	
N3, n (%)	10 (3.2)	2 (1.1)	8 (5.8)	
Number of excised lymph nodes (Median) (IQR)	16 (12–23)	15 (12–23)	17 (12–24)	0.45^m^
Number of metastatic lymph nodes (Median) (IQR)	0 (0–1)	0 (0–0)	0 (0–2)	**< 0.001** ^**m**^
Presence of carcinoma in situ, n (%)	102 (32.2)	57 (32)	45 (32.4)	0.947^c^
Presence of prostate invasion, n (%)	17 (5.4)	4 (2.2)	13 (9.4)	**0.005** ^**c**^
Concomitant primary prostate cancer, n (%)	98 (30.9)	59 (33.1)	39 (28.1)	0.331^c^
Gleason score 3 + 3, n	76	46	30	
Gleason score 3 + 4, n	19	10	9	
Gleason score 4 + 3, n	2	2	0	
Gleason score 4 + 4, n	1	1	0	
Follow-up period (months) (Median) (IQR)	18 (8-31.4)	24.1 (12-36.7)	12 (6.5–21.7)	**< 0.001** ^**m**^

*BMI* Body Mass Index, *IQR* Interquartile Range, *ASA* American Society of Anesthesiologists, *WBC* White Blood Cell, *HT* Hypertension, *DM* Diabetes Mellitus, *CKD* Chronic Kidney Disease, *CVD* Cerebrovascular Disease, *COPD* Chronic Obstructive Pulmonary Disease, *TPN* Total Parenteral Nutrition, *MIBC* Muscle Invasive Bladder Cancer, *NMIBC* Non-muscle Invasive Bladder Cancer, ^t^ Independent Sample T Test, ^m^ Mann Whitney U Test, ^k^ Chi-Square Test, ^f^ Fisher’s Exact Test

Bold *p* values indicate statistical significance

**Table 2:** Analysis of the types and prevalence of variant histologies in pathological specimens from patients treated with radical cystectomy with ileal conduit urinary diversion for urothelial carcinoma

	Total (*n* = 317)
Variant Histology	*n* (%)
Sarcomatoid Urothelial Carcinoma	13 (4.1)
Squamous Differentiation	80 (25.2)
Glandular Differentiation	20 (6.3)
Micropapillary Urothelial Carcinoma	24 (7.6)
Nested Variant	11 (3.5)
Neuroendocrine/Small Cell Differentiation	4 (1.3)
Plazmacytoid Urothelial Carcinoma	8 (2.5)
Other	17 (5.4)

* The presence of multiple histological variants within the same pathological specimen is possible

Patients were divided into two groups based on the presence of VH. In the variant patient group, the presence of hydronephrosis, clinical lymph node positivity, WBC level, pathological N stage, number of metastatic lymph nodes and presence of prostate invasion were higher compared to the pure UC patient group (respectively *p* = 0.034, *p* = 0.001, *p* < 0.001, *p* = 0.001, *p* < 0.001, *p* = 0.005). The rate of intravesical treatment history and follow-up duration were lower (*p* = 0.031, *p* < 0.001, respectively). The demographic, preoperative, intraoperative and postoperative data of the pure UC and VH patient groups and their comparative analysis are shown in Table 1.

ROC curve was produced with a 95% CI for demonstrating the predictive value of WBC for VH and the cut-off point was determined as was 7695 g/dl (AUC = 0.635, CI: 0.574–0.697; *p* < 0.001).

Multivariate logistic regression analysis was applied to determine factors associated with VH. Presence of hydronephrosis (OR = 1.638; 95% CI = 1.02–2.63; *p* = 0.041), clinical lymph node positivity (OR = 2.301; 95% CI = 1.325–3.996; *p* = 0.003) and WBC > 7695 per g/dl (OR = 2.452; 95% CI = 1.513–3.972; *p* < 0.001) were defined as factors associated with VH (Table 3).

**Table 3:** Multivariate logistic regression analysis of factors associated with the presence of variant histology in patients treated with radical cystectomy with ileal conduit urinary diversion for urothelial carcinoma

	OR (95% CI)	*p*
Presence of hydronephrosis	1.638 (1.02–2.63)	0.041
Clinical lymph node positivity	2.301 (1.325–3.996)	**0.003**
WBC > 7695 per g/dl	2.452 (1.513–3.972)	**< 0.001**

*CI* Confidence Interval, *WBC* White Blood Cell

Bold values indicate statistical significance

**Fig. 1:**
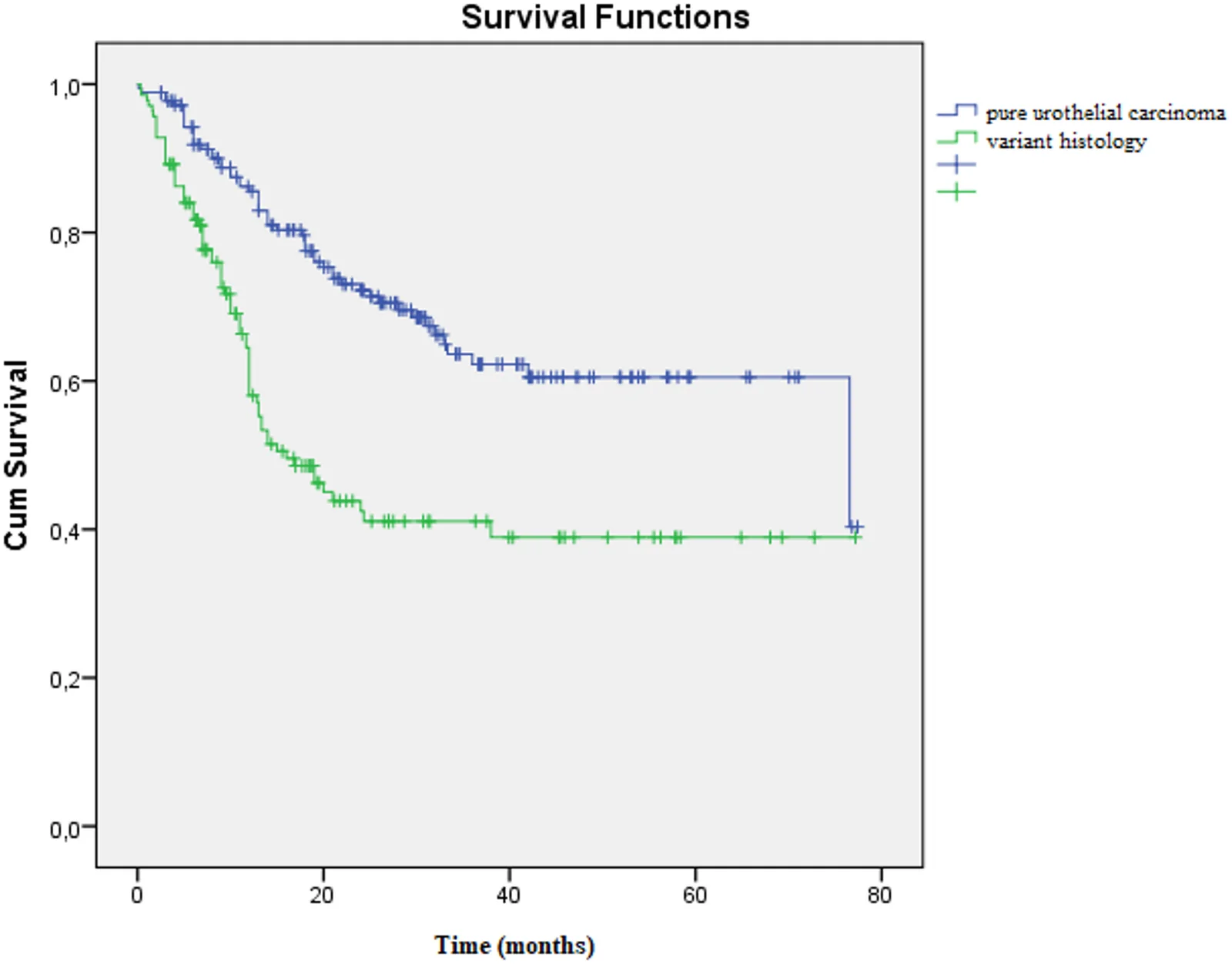
Kaplan–Meier estimate of overall survival for patients with pure urothelial carcinoma and variant histologies treated with radical cystectomy with ileal conduit urinary diversion

The median follow-up period was 18 months (IQR = 8–31.4 months). The 1- and 3-year OS rates were 85.6% and 62.2% for patients with pure UC and those with VH were 58% and 38.9%, respectively. The mean OS for patients with pure UC and those with VH were 53.8 months (95% CI = 48.644–58.886) and 36.8 months (95% CI = 30.433–43.068), respectively. Kaplan–Meier survival analysis, depicted in Fig. 1, illustrates a statistically significant reduction in OS among patients harboring VH compared to those with pure UC following RC and ileal conduit urinary diversion (log-rank test, *p* < 0.001).

**Fig. 2:**
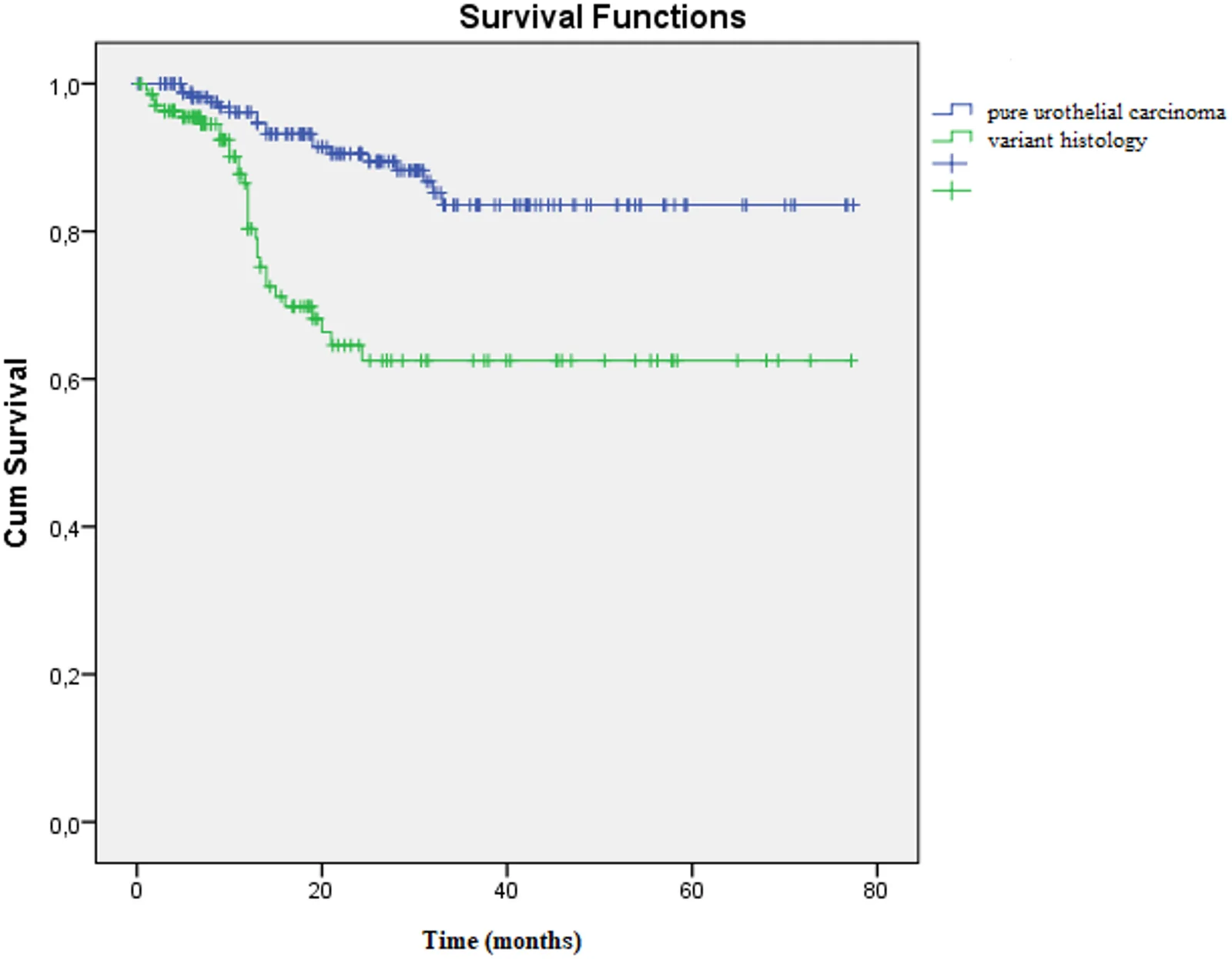
Kaplan–Meier estimate of cancer specific survival for patients with pure urothelial carcinoma and variant histologies treated with radical cystectomy with ileal conduit urinary diversion

The 1- and 2-year CSS rates were 94.7% and 89.5% for patients with pure UC and those with VH were 80.3% and 62.5%, respectively. The mean CSS were 68 months (95% CI = 63.888–72.086) for the pure UC cohort and 53 months (95% CI = 45.964–60.034) for the VH cohort. Figure 2 demonstrates the Kaplan–Meier estimates of CSS, which similarly indicate a significantly poorer prognosis in patients with VH (log-rank test, *p* < 0.001).

Multivariable Cox regression analysis was performed to identify factors associated with OS and CSS. Low serum hemoglobin level (HR = 0.893; 95% CI = 0.815–0.98; *p* = 0.017), pathological stage ≥ pT3 (HR = 2.177; 95% CI = 1.397–3.392; *p* = 0.001), higher number of metastatic lymph nodes (HR = 1.046; 95% CI = 1.001–1.093; *p* = 0.045), presence of prostate invasion (HR = 1.974; 95% CI = 1.068–3.651; *p* = 0.03) and presence of variant histology (HR = 1.624; 95% CI = 1.094–2.411; *p* = 0.016) in the final pathology were found as independent predictors of reduced OS (Table 4). Pathological stage ≥ pT3 (HR = 3.889; 95% CI = 1.849–8.179; *p* < 0.001), higher number of metastatic lymph nodes (HR = 1.066; 95% CI = 1.002–1.135; *p* = 0.044), presence of CIS (HR = 2.122; 95% CI = 1.042–4.321; *p* = 0.038) and presence of variant histology (HR = 1.945; 95% CI = 1.043–3.626; *p* = 0.036) in the final pathology were found as independent predictors of reduced CSS (Table 5).

**Table 4:** Evaluation of the effects of demographic, preoperative, intraoperative and postoperative characteristics of patients undergoing radical cystectomy with ileal conduit diversion for urothelial carcinoma on overall survival by Cox regression analysis

Univariate			Multivariate		
	HR (95% CI)	*p*		HR (95% CI)	*p*
Age (per year)	1.024 (0.999–1.05)	0.057	Age (per year)	1.024 (0.999–1.049)	0.061
Male gender	0.984 (0.553–1.748)	0.955			
BMI (per kg/m^2^)	0.954 (0.908–1.002)	0.061	BMI (per kg/m^2^)	0.97 (0.92–1.022)	0.25
Previous surgery history	1.092 (0.682–1.748)	0.715			
Intravesical treatment history	0.965 (0.543–1.716)	0.903			
Neoadjuvant chemotherapy history	0.965 (0.618–1.507)	0.874			
Presence of concomitant malignancy	0.878 (0.357–2.157)	0.777			
Presence of hydronephrosis	1.526 (1.07–2.176)	**0.02**	Presence of hydronephrosis	1.068 (0.727–1.57)	0.738
ASA score (per score)	1.424 (0.998–2.031)	0.051	ASA score (per score)	1.303 (0.9-1.887)	0.161
Clinical T2 stage	1.458 (0.957–2.223)	0.08			
Clinical lymph node positivity	1.644 (1.118–2.418)	**0.011**			
Presence of distant metastasis	1.941 (1.092–3.451)	**0.024**			
Hemoglobin (per g/dl)	0.872 (0.794–0.957)	**0.004**	Hemoglobin (per g/dl)	0.893 (0.815–0.98)	**0.017**
WBC (per g/dl)	1.005 (0.932–1.013)	0.115			
Serum creatine (per mg/dl)	1.356 (0.937–1.96)	0.106			
Serum albumine (per g/L)	0.964 (0.93-1)	**0.048**	Serum albumine (per g/L)	0.967 (0.928–1.008)	0.109
Presence of complication	1.386 (0.973–1.975)	0.07			
Pathological stage (≥ pT3)	3.162 (2.12–4.716)	**< 0.001**	Pathological stage (≥ pT3)	2.177 (1.397–3.392)	**0.001**
Number of metastatic lymph nodes	1.079 (1.039–1.121)	**< 0.001**	Number of metastatic lymph nodes	1.046 (1.001–1.093)	**0.045**
Presence of carcinoma in situ	1.018 (0.696–1.49)	0.926			
Presence of prostate invasion	3.528 (1.982–6.28)	**< 0.001**	Presence of prostate invasion	1.974 (1.068–3.651)	**0.03**
Presence of variant histology	2.344 (1.637–3.356)	**< 0.001**	Presence of variant histology	1.624 (1.094–2.411)	**0.016**

*BMI* Body mass index, *ASA* American Society of Anesthesiologists, *NAC* Neoadjuvant chemotherapy, *WBC* White blood cell

Bold values indicate statistical significance

**Table 5:** Evaluation of the effects of demographic, preoperative, intraoperative and postoperative characteristics of patients undergoing radical cystectomy with ileal conduit diversion for urothelial carcinoma on cancer-specific survival survival by Cox regression analysis

Univariate			Multivariate		
	HR (95% CI)	*p*		HR (95% CI)	*p*
Age (per year)	1.001 (0.966–1.038)	0.943			
Male gender	1.865 (0.905–3.845)	0.091			
BMI (per kg/m^2^)	1.005 (0.932–1.083)	0.895			
Previous surgery history	1.548 (0.79–3.032)	0.203			
Intravesical treatment history	1.56 (0.73–3.332)	0.251			
Neoadjuvant chemotherapy history	1.283 (0.669–2.46)	0.454			
Presence of concomitant malignancy	1.375 (0.427–4.43)	0.593			
Presence of hydronephrosis	1.858 (1.059–3.259)	**0.031**	Presence of hydronephrosis	1.379 (0.772–2.463)	0.277
ASA score (per score)	1.384 (0.788–2.43)	0.258			
Clinical T2 stage	1.327 (0.692–2.546)	0.394			
Clinical lymph node positivity	2.073 (1.15–3.734)	**0.015**			
Presence of distant metastasis	2.742 (1.231–6.107)	**0.014**			
Hemoglobin (per g/dl)	0.935 (0.807–1.084)	0.373			
WBC (per g/dl)	1 (1-1.001)	0.055			
Serum creatine (per mg/dl)	1.335 (0.746–2.388)	0.331			
Serum albumine (per g/L)	0.992 (0.934–1.054)	0.803			
Presence of complication	1.08 (0.615–1.896)	0.789			
Pathological stage (≥ pT3)	5.156 (2.561–10.378)	**< 0.001**	Pathological stage (≥ pT3)	3.889 (1.849–8.179)	**< 0.001**
Number of metastatic lymph nodes	1.095 (1.038–1.154)	**0.001**	Number of metastatic lymph nodes	1.066 (1.002–1.135)	**0.044**
Presence of carcinoma in situ	1.805 (0.901–3.617)	0.096	Presence of carcinoma in situ	2.122 (1.042–4.321)	**0.038**
Presence of prostate invasion	1.323 (0.321–5.455)	0.699			
Presence of variant histology	3.406 (1.898–6.111)	**< 0.001**	Presence of variant histology	1.945 (1.043–3.626)	**0.036**

*BMI* Body mass index, *ASA* American Society of Anesthesiologists, *NAC* Neoadjuvant chemotherapy, *WBC* White blood cell

Bold values indicate statistical significance

## Discussion

In the current study, we collected and processed the preoperative, postoperative, and survival data of 317 patients who underwent RC with ileal conduit urinary diversion. The focus of the study was to evaluate the preoperative and postoperative characteristics of the cases bearing VH in the RC specimen and the impact of VH on patient survival. Statistical analysis showed that patients with VH had more locally advanced disease (expressed by increased rates of tumor infiltration-related hydronephrosis, clinical and pathological lymph node positivity, prostate invasion) and reduced survival (expressed by the faster decline of Kaplan-Meier curves in CSS and OS plots). Interestingly, VH was significantly and independently associated not only with local extension (hydronephrosis, clinical lymph node positivity) but also with increased WBC count, which showed the strongest correlation with VH.

To compare our findings with recent reports on the same topic, we performed a thorough search of the available bibliography and found that the respective results are inconsistent. According to a meta-analysis by Mori et al., VH is indeed associated with increased rates of disease relapse after RC and consequently worse OS and CSS rates. Of note, among different variants, micropapillary, plasmacytoid, and small cell subtypes were particularly correlated with reduced survival [13]. Similarly, Naspro et al. showed that the presence of VH in a muscle-invasive bladder cancer (MIBC) patient cohort was accompanied by increased rates of locally advanced disease (tumour stage ≥ pT3), lymph node metastasis, and cancer-related death. In this study, micropapillary, sarcomatoid, and small cell subtypes were mainly correlated with shortened survival [14]. In 2021, Minato et al. studied the histological and survival data of 150 MIBC patients who were treated with RC and found that VH was significantly and independently associated with disease recurrence and mortality in the patient subgroup with VH presence ≥ 80% of the examined specimen [15]. More recently, Lee et al. processed the clinical and survival data of 299 patients who were diagnosed with urothelial BCa and underwent RC. In the above cohort, 55 patients had VH in their histologic specimen, and among them, 20 patients had aggressive VH (micropapillary, plasmacytoid, or sarcomatoid). The latest subcohort demonstrated a significant reduction of OS and progression-free survival (PFS) [16].

Regarding the prediction of VH presence in the pre-cystectomy setting, our literature search found only one report by Prijovic et al. In this study, patients with VH were significantly older, they had significantly more leucocytes, neutrophils, and more frequently hydronephrosis, locally advanced disease, and infiltrated lymph nodes. However, neutrophil count was the only significant predictor of VH in the multivariate analysis [17].

Despite the lack of evidence on the association between WBC (or neutrophils) with VH, we found many studies that correlated WBC (either as alone factor, or as a factor of a mathematical formula) with patient survival in almost every clinical scenario of the urothelial BCa. In 2021, Katayama et al. processed the preoperative and postoperative data of 1117 non-muscle invasive bladder cancer (NMIBC) patients and found that blood-based systemic immune-inflammation index (SII, an indicator calculated with the neutrophil, platelet, and leucocyte count) was a significant and independent predictor of PFS and CSS [18]. Another study by von Deimling et al. was conducted in a patient group planned for NAC before RC and demonstrated that a high neutrophil-to-lymphocyte ratio (NLR) was significantly associated with poor response to the chemotherapeutic regimen [19]. In the metastatic BCa scenario, a study by Degerli et al. showed that both pre-immunotherapy and post-immunotherapy NLR values were significant predictors of patient survival [20]. Recently, a prospective study by Pyrgidis et al. evaluated several preoperative and perioperative risk factors of 1817 patients who underwent RC, and reported that preoperative leucocytosis was an independent and significant predictor of patient survival [21].

The above evidence suggests that an increased WBC (or neutrophils, which comprise the main component of WBC) seems to have cancer-promoting effects, which can explain its association with decreased survival in patients with MIBC, locally advanced, or metastatic disease. The above suggestion is supported by evidence from the basic research, since many studies report that neutrophils can promote neoangiogenesis by releasing pro-angiogenic factors in the tumor microenvironment [22]. Similarly, neutrophils seem to play a role in tumor-induced lymphangiogenesis through migrating to the existing lymphatic network and secreting lymphotropic factors, which in turn trigger the construction of new lymphatic vessels [23]. Nevertheless, the discriminatory performance of WBC, as reflected by an AUC of 0.635, was limited in the current study. Therefore, WBC should not be considered a clinically robust predictive biomarker on its own, but rather as an adjunctive indicator that may gain value when combined with other clinical or pathological parameters.

Regarding the etiological relationship among VH, reduced survival, locally advanced disease (hydronephrosis, clinical/ pathological lymph node positivity), from our results and the available evidence in the urological literature there are strong indications that at least specific VH entities are characterized by aggressive biological behavior, which is reflected in an increased tendency for local extension and distant metastases. Moreover, VH is strongly correlated with WBC, yet it seems plausible that their common etiology is again the aggressive tumor biology, which histologically is detectable as VH and at the blood level is reflected in increased WBC. To further support the above findings, prospective trials with stratification of adequate patients and quantification of the VH component are needed to unveil the exact effect of each VH subtype and the exact etiology among the recorded parameters. Another option for the production of robust evidence is the application of molecular instead of histological criteria, since the former depict more precisely the tumour biology.

In the current study, presence on variant histology was found as associated with worse OS and CSS. Our findings are in line with previous reports indicating that variant histology represents a more biologically aggressive disease subtype. Laymon et al. compared oncologic outcomes between squamous cell carcinoma and urothelial carcinoma with squamous differentiation after radical cystectomy and found that the presence of squamous differentiation was associated with significantly worse survival compared to pure urothelial carcinoma [24]. This supports our observation that variant histology is linked to adverse pathological features and poorer oncologic outcomes.

The present study has several limitations that should be acknowledged. It was designed as a retrospective, single-institution analysis with a small sample size, which may introduce selection bias and limit the generalizability of the findings. Future multicenter prospective studies are warranted to better clarify the biological behavior of VH and their response to neoadjuvant and adjuvant systemic therapies. So, direct impact of the results on current practice remains limited, although potential long-term interest exists. All consecutive patients were initially reviewed. Exclusion of those with incomplete records could have introduced a degree of selection bias, as missing data may not have been random. The use of hospital records may have led to missing or incomplete data, particularly regarding perioperative variables and follow-up information. All radical cystectomies were performed by experienced surgeons. However, minor heterogeneity in surgical technique and perioperative management cannot be excluded. Perioperative chemotherapy (particularly NAC) was evenly distributed between VH and pure UC groups and did not show a significant effect on OS or CSS in multivariable analysis. This lack of association might be related to the relatively short follow-up period, which may not have allowed sufficient time for chemotherapy-related survival differences to emerge. Although pathological evaluations were conducted by experienced uropathologists, the retrospective design of the study meant that multiple pathologists were involved in the assessments, which may have introduced interobserver variability in the identification and classification of variant histology. Stage-for-stage outcome analyses were not performed, as the relatively limited sample size and number of events within each pathological stage subgroup would likely result in insufficient statistical power and unreliable estimates. The lack of external validation and molecular characterization of variant histologies limits the ability to draw definitive causal inferences. Therefore, the results should be interpreted with caution. The median follow-up duration of 18 months is relatively short for radical cystectomy cohorts, and therefore, the 3-year overall and cancer-specific survival rates should be interpreted with caution. Another important consideration is the lack of consistency in the definition of VH across studies. While our classification was based on the WHO criteria, some series do not include squamous or glandular differentiation within the definition of variant histology. This heterogeneity in classification may limit direct comparison of results across studies. A further limitation of this study is that we could not stratify variant histology into specific subtypes. Heterogenous group of VH may behave differently with clinical outcomes and plasmacytoid, small cell and nested variant may be very different tumor biologies and clinical behaviors. The relatively small number of patients in each variant group and the coexistence of multiple variants within some specimens would have compromised statistical validity. Therefore, VH was analyzed as a single entity, and future larger studies are needed to enable reliable subtype-based comparisons. While the role of NAC in VH was evaluated in several studies, the rapid development of novel systemic therapies, including immunotherapy and targeted agents, highlights the need for future research focusing on their efficacy in this heterogeneous patient population.

## Conclusion

In the current study, we collected and processed the preoperative, perioperative, and survival data of a patient cohort that underwent RC for high-risk NMIBC or MIBC to investigate the relationship of VH with the oncological outcomes. Our results show that VH is associated with shortened CSS and OS. Moreover, VH is strongly correlated with specific preoperative parameters, which include hydronephrosis, clinical lymphadenopathy, and increased WBC. The above factors may be applied as factors associated with VH in respective predictive models. Nevertheless, given the relatively short follow-up period, our survival results should be interpreted with caution and validated in studies with longer observation periods.

## Data Availability

The datasets generated and/or analysed during the current study are available in Figshare Repository at https://figshare.com/s/c039125c55fd2d0d0010.
